# Social isolation suppresses actin dynamics and synaptic plasticity through ADF/cofilin inactivation in the developing rat barrel cortex

**DOI:** 10.1038/s41598-017-08849-3

**Published:** 2017-08-16

**Authors:** Hirobumi Tada, Tomoyuki Miyazaki, Kiwamu Takemoto, Susumu Jitsuki, Waki Nakajima, Mayu Koide, Naoko Yamamoto, Akiko Taguchi, Honami Kawai, Kasane Komiya, Kumiko Suyama, Hiroki Abe, Akane Sano, Takuya Takahashi

**Affiliations:** 1 Department of Physiology, Yokohama City University Graduate School of Medicine, Yokohama, 236-0004 Japan; 2 Department of Integrative Aging Neuroscience, National Center for Geriatrics and Gerontology, Aichi, 474-8511 Japan; 3 JST, PRESTO, Saitama, 332-0012 Japan

## Abstract

Exposure to a stressful environment early in life can cause psychiatric disorders by disrupting circuit formation. Actin plays central roles in regulating neuronal structure and protein trafficking. We have recently reported that neonatal isolation inactivated ADF/cofilin, the actin depolymerizing factor, resulted in a reduced actin dynamics at spines and an attenuation of synaptic α-amino-3-hydroxy-5-methyl-4-isoxazole propionic acid (AMPA) receptor delivery in the juvenile rat medial prefrontal cortex (mPFC), leading to altered social behaviours. Here, we investigated the impact of neonatal social isolation in the developing rat barrel cortex. Similar to the mPFC study, we detected an increase in stable actin fraction in spines and this resulted in a decreased synaptic AMPA receptor delivery. Thus, we conclude that early life social isolation affects multiple cortical areas with common molecular changes.

## Introduction

The development of functional circuits in the cortex is crucial for cognitive functions, and their dysfunction may lead to a variety of psychiatric disorders^1, 2^. Sensory misprocessing potentially caused by abnormal circuit functioning in the somatosensory cortex is a prominent symptom of mental illness^3^.

Prolonged exposure to stressful situations early in life can affect the formation of neural circuits in the cortex leading to cognitive dysfunctions^4–6^. Synaptic plasticity driven by experience plays central roles in the establishment of neural circuits^4, 6–22^. Thus, elucidating the molecular and cellular mechanisms underlying experience-dependent synaptic reorganization in the cortex following chronic neonatal stress is a prerequisite for understanding how neonatal maltreatment can induce mental disorders.

A number of studies have examined the molecular events occurring at synapses during the development of experience-driven neural plasticity. The synaptic insertion of glutamate α-amino-3-hydroxy-5-methyl-4-isoxazole propionic acid (AMPA) receptors is one crucial mechanism that underlies this process^7–14, 19–22^. In young rodents, whisker experience drives GluA1 subunit-containing AMPA receptors into synapses in the developing barrel cortex^4, 7^. In addition, learning experience delivers GluA1 into synapses of the amygdala and hippocampus, and is required for memory formation^10–12^.

Actin, the principal cytoskeletal component of synapses, is enriched in postsynaptic spines^23–25^. Actin also regulates the assembly of postsynaptic proteins including AMPA receptors^23, 24, 26–28^. The dynamics of actin are regulated by a number of proteins. For example, the actin-depolymerizing factor (ADF)/cofilin family of actin-binding proteins plays an essential role in the turnover of actin fibres^29^. Moreover, ADF/cofilin mediates AMPA receptor trafficking during synaptic plasticity, demonstrating thus the importance of actin dynamics for AMPA receptor delivery^26^.

We recently reported that neonatal social isolation inactivated ADF/cofilin and led to an increase in stable actin fractions at the dendritic spines in the juvenile rat medial prefrontal cortex (mPFC). Moreover, isolation-induced increase of stable actin fractions disrupted the experience-driven synaptic delivery of AMPA receptors and resulted in altered social behaviours^5^. Although there is strong correlation between mPFC and somatosensory cortex to develop social behaviours^30–32^, it is unclear how social isolation affect actin dynamics and regulate synaptic delivery of AMPA receptors in the somatosensory cortex.

Here, we found that neonatal social isolation induced the same molecular events at spines of the developing rat barrel cortex, implying the wide effects of neonatal isolation throughout the cortex.

## Results

### Neonatal isolation increases the fraction of stable actin in the developing rat barrel cortex

We recently reported that neonatal isolation affects actin dynamics at spines and synaptic AMPA receptor delivery in the juvenile mPFC^5^. To examine whether such events have a widespread occurrence in the cortex, we investigated the impact of early social isolation on developing rat barrel cortex. We isolated male pups from their mother and siblings for six hours per day for five days starting at postnatal day 7 (P7). Using this stress paradigm, we previously reported a long-lasting increase in corticosterone levels, a stress-induced glucocorticoid hormone, and an attenuation in experience-driven AMPA receptor delivery to synapses in layers 4 to 2/3 in the rat barrel cortex at P12–P14^4^. Since actin dynamics regulate AMPA receptor trafficking at spines^26^, we examined whether early isolation affects actin dynamics. We introduced an expression vector for Green Fluorescent Protein (GFP)-tagged actin by *in utero* electroporation into the cortical area that develops into the barrel cortex. We subsequently subjected the resulting pups to neonatal isolation from P7-P11. At P14, we prepared acute brain slices and performed Fluorescence Recovery After Photobleaching analysis (FRAP)^5^.

In the FRAP analysis, an individual spine in layer 2/3 of the barrel cortex was photobleached using high intensity laser illumination with a two-photon laser-scanning microscope (excitation wavelength of 910 nm). The time course of the subsequent fluorescence recovery in the photobleached spine was used to evaluate actin dynamics. Actin turnover depends on polymerization at the barbed end and depolymerization at the pointed end^25^. Typically, in the case of proteins that diffuse freely, a rapid and complete recovery of the photobleached fluorescence is observed. The existence of stable actin filaments with reduced turnover results in the increase of an unrecoverable fraction during FRAP analysis.

**Figure 1:**
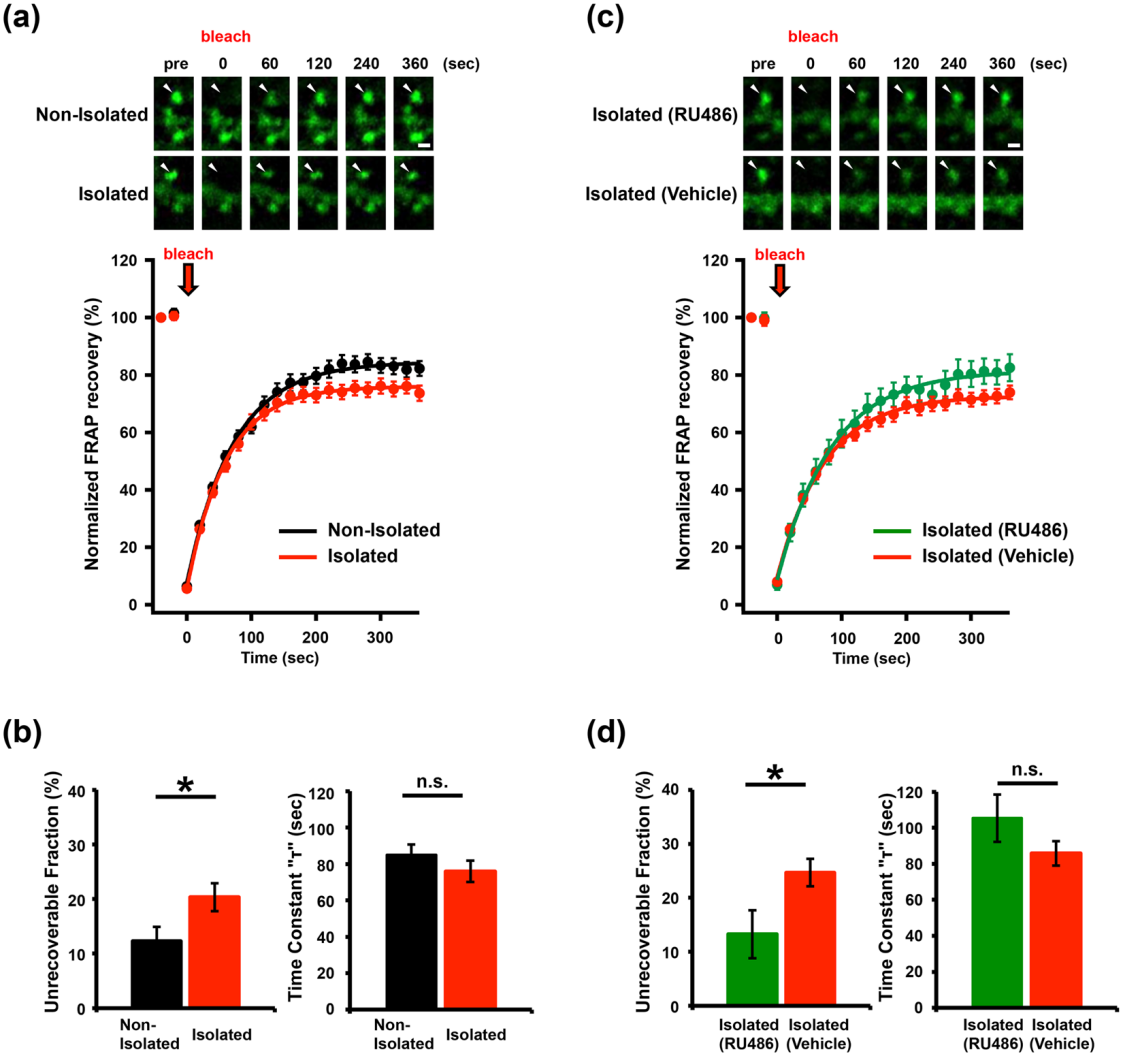
Social isolation alters actin dynamics via glucocorticoid signalling. (**a**) FRAP analysis of GFP-actin in spines from the barrel cortex of isolated or non-isolated rats. Representative images and graphs depicting the recovery of GFP-actin signals before and after spine photobleaching are shown. (**b**) Recovery time constant and quantification of the unrecoverable fraction of spines from isolated or non-isolated rats. The unrecoverable actin fraction in the spines of the barrel cortex was increased in isolated rats (n = 54) compared to non-isolated controls (n = 57). (**c,d**) The isolation-induced increase in the unrecoverable fraction was blocked by the administration of RU486 (n = 22) during isolation, but not by treatment with vehicle (n = 39). Representative images shown in the figures were Gaussian- and unsharp mask-filtered. Arrowheads indicate dendritic spines. Scale bar represents 1 µm. *P < 0.05 (Unpaired Student’s t-test). Error bars represent SEM.

We detected a prominent increase in the unrecoverable fraction of GFP-actin in the photobleached spines obtained from socially-isolated rats compared to non-isolated controls (Fig. 1a,b). This observation indicates that early social isolation increases the stable immobilized actin fraction. We observed no difference in the time constant (τ) of fluorescence recovery between isolated and non-isolated rats (Fig. 1a,b). This finding suggested that there could exist two fractions of actin fibres: one that is unaffected by neonatal social isolation and maintains a normal turnover, and another that is modified following isolation and ceases dynamic turnover resulting in the increase in unrecoverable fraction after rapid photobleaching.

We previously reported that this social isolation procedure increases the level of circulating free corticosterone, a major stress hormone in rodents^4^. To examine if the neonatal isolation-induced increase in the stable actin fraction is mediated by glucocorticoid signalling, we injected animals with RU486, an antagonist of the glucocorticoid receptor (GR), during the social isolation procedure followed by FRAP analysis of GFP-actin-expressing spines. We found that the unrecoverable fraction of photobleached GFP-actin in spines from isolated rats treated with RU486 was significantly smaller than that from isolated rats that did not receive RU486, and was comparable to that of non-isolated controls (Fig. 1c,d: compare with Fig. 1a,b). Therefore, we concluded that the isolation-induced increase in the stable actin fraction was dependent on glucocorticoid signalling.

### Inactivation of ADF/cofilin mediates the social-isolation-induced increase in the stable actin fraction in the developing rat barrel cortex

**Figure 2:**
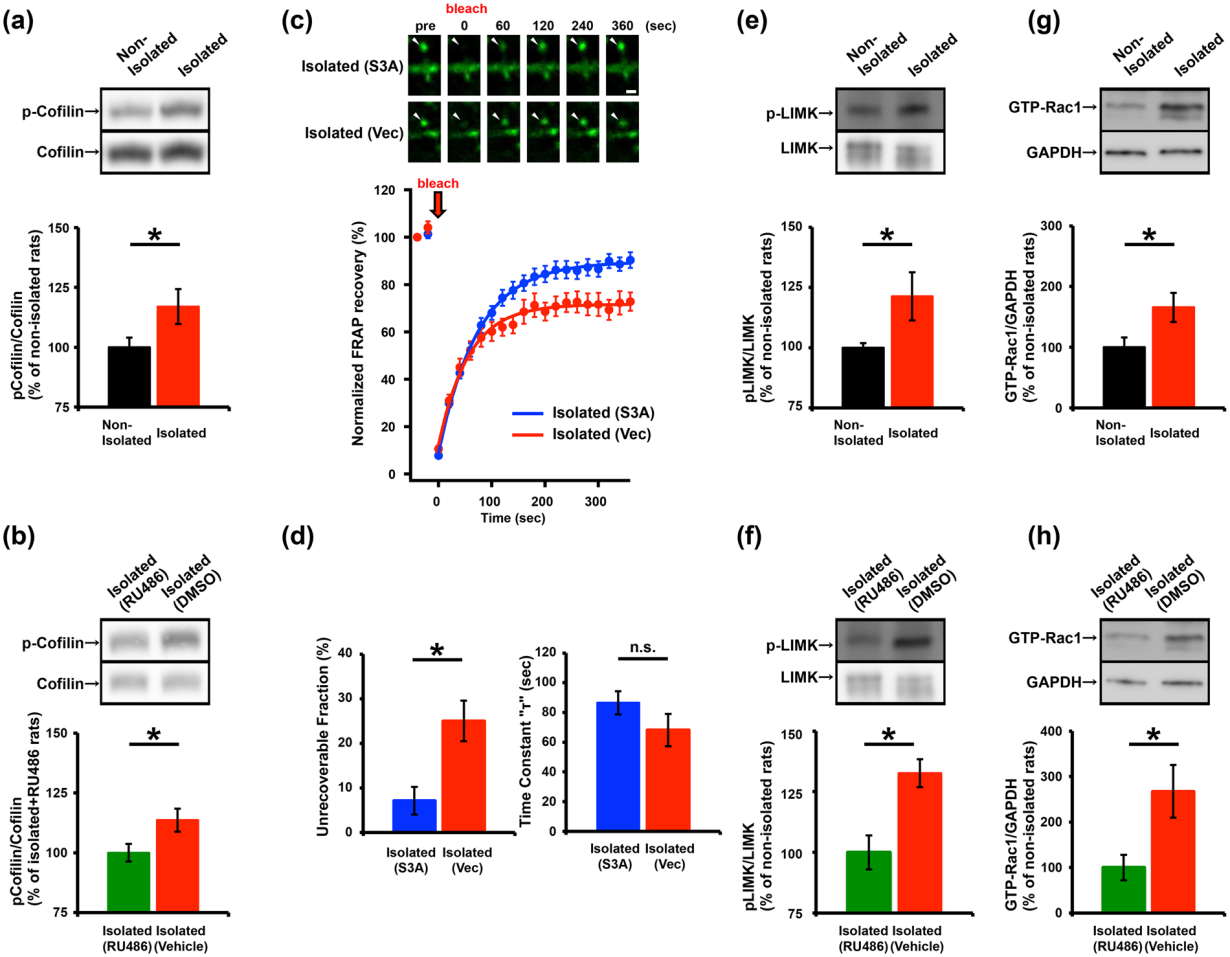
ADF/cofilin inactivation via Rac1-LIM kinase signalling mediates the increase in stable actin at spines of the barrel cortex in socially isolated rats. Representative western blots of the barrel cortex synaptoneurosomes from non-isolated or isolated rats (**a,e**, and **f**), and isolated rats with or without RU486 treatment (**b**). (**a**) Phosphorylation of ADF/cofilin (Ser3) was increased in the barrel cortex of isolated rats (n = 34) compared to non-isolated rats (n = 34). (**b**) The increased phosphorylation of ADF/cofilin in isolated rats was blocked by the administration of RU486 (n = 14) during isolation but not by treatment with vehicle (n = 13). (**c**) FRAP analysis showing the effect of ADF/cofilin phospho-mutant (S3A) expression on actin dynamics in the spines of the barrel cortex of isolated rats. Representative images and graphs depicting the recovery of the GFP-actin signal before and after photobleaching at spines expressing GFP-actin with RFP-S3A or RFP alone (control) in isolated rats are shown. Representative images shown in the figures were Gaussian- and unsharp mask-filtered. Arrowheads indicate dendritic spines. Scale bar represents 1 µm. (**d**) Recovery time constant and quantification of the unrecoverable fraction of spines expressing the ADF/cofilin phospho-mutant in isolated rats. The increased unrecoverable actin fraction in isolated rats was prevented in the spines expressing RFP-S3A (n = 27) but not in control spines expressing RFP (n = 21). (**e**) Phosphorylation of LIM kinase (LIMK) was increased in the barrel cortex of isolated rats (n = 5) compared with the non-isolated controls (n = 7). (**f**) The increased phosphorylation of LIMK in isolated rats was blocked by the administration of RU486 (n = 7) during isolation but not by treatment with vehicle (n = 8). (**g**) Rac1 activity (GTP-Rac1) was increased in the barrel cortex of isolated rats (n = 9) compared with the non-isolated controls (n = 9). (**h**) The increased GTP-Rac1 in isolated rats was blocked by the administration of RU486 (n = 8) during isolation but not by treatment with vehicle (n = 5). The images shown were the cropped blots for the corresponding proteins. The original uncropped images were shown in Supplementary Figure 1. *P < 0.05 (Unpaired Student’s t-test). Error bars represent SEM.

A number of proteins regulate actin dynamics. ADF/cofilin is an actin-depolymerizing factor and is essential for the turnover of actin fibres^26, 29^. The ADF/cofilin is inactivated or activated by phosphorylation or dephosphorylation of its serine-3 (Ser3) residue, respectively^26^. To determine whether neonatal isolation stress alters the activity of ADF/cofilin, we measured Ser3 phosphorylation in ADF/cofilin expressed in the barrel cortex of non-isolated controls, isolated, and isolated RU486 -treated rats. Synaptoneurosome fractions isolated from the barrel cortex of each of the treatments groups were prepared at P11, then examined for the Ser3 phosphorylation of ADF/cofilin. We detected elevated ADF/cofilin phosphorylation at Ser3 in isolated rats compared to non-isolated controls and isolated RU486-treated rats (Fig. 2a,b). These observations indicate that neonatal isolation suppresses ADF/cofilin activity via glucocorticoid signalling in the developing barrel cortex as was observed in the juvenile mPFC^5^.

We next asked if ADF/cofilin inactivation mediates the isolation-induced increase in the stable actin fraction at spines in the barrel cortex. We introduced expression vectors for GFP-actin and a red fluorescent protein (RFP)-tagged ADF/cofilin Ser3A (a constitutively active form in which Ser3 is mutated to alanine) by *in utero* electroporation^26^. We then exposed the pups to the isolation procedure from P7-P11 and prepared acute brain slices for FRAP analysis at P14. We found that the unrecoverable fraction of GFP-actin after photobleaching at spines in layer 2/3 of the barrel cortex of isolated rats expressing Ser3A was comparable to that of the non-isolated controls (Figs 1a,b and 2c,d). Moreover, isolated rats exhibited a significantly larger unrecoverable fraction of GFP-actin at spines after rapid photobleaching than isolated rats expressing Ser3A (Fig. 2c,d). These results indicate that the isolation-induced increase in the stable actin fraction is mediated by ADF/cofilin inactivation.

The activity of cofilin is regulated by LIM kinase, which phosphorylates Ser3 of ADF/cofilin resulting in its inactivation. We next examined the activity of LIM kinase in the developing barrel cortex of isolated and non-isolated rats. The synaptoneurosome fractions from the barrel cortex of these rats were prepared at P11. The LIM kinase phosphorylation at threonine 508 (T508), an indicator of LIM kinase activity, was then assessed^33, 34^. We found an increased T508 phosphorylation of LIM kinase in the isolated rats compared to non-isolated controls and isolated RU486-treated rats (Fig. 2e,f). Furthermore, we compared Rac1 activation between isolated and non-isolated rats given its reported involvement in LIM kinase activity^35^. The synaptoneurosome fraction prepared from isolated rats exhibited an increase in the GTP-bound activated form of Rac1 compared to non-isolated rats and isolated RU486-treated rats (Fig. 2g,h). Together, these findings indicate that neonatal isolation activates Rac1 leading to LIM kinase activation and ADF/cofilin inactivation via Ser3 phosphorylation.

### ADF/cofilin inactivation mediates the social-isolation-induced suppression of experience-driven synaptic delivery of the AMPA receptor in the developing barrel cortex

**Figure 3:**
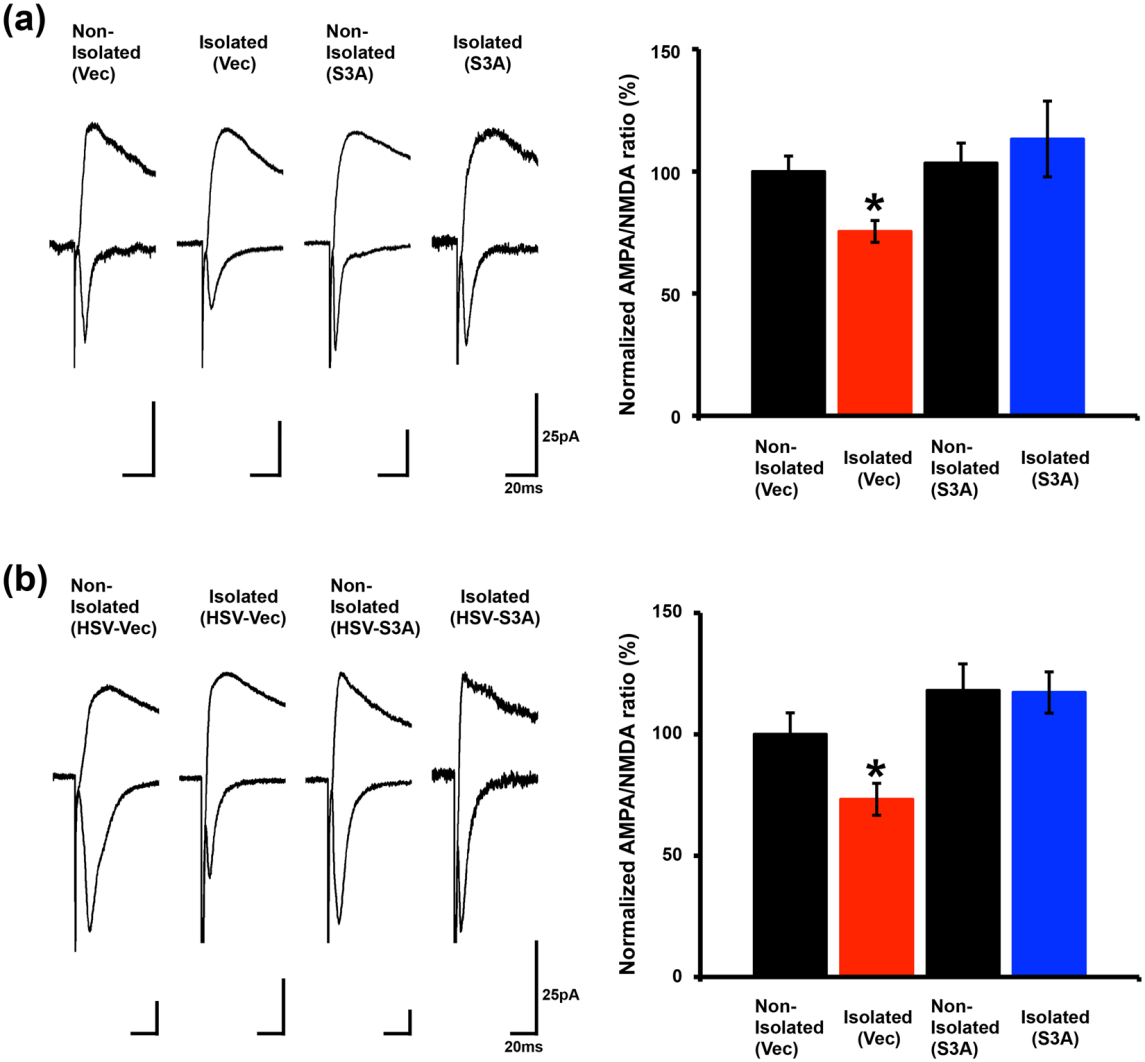
Expression of the constitutively active ADF/cofilin phospho-mutant (S3A) leads to the recovery of experience-driven synaptic AMPA receptor delivery in the barrel cortex of socially isolated rats. (**a**) Representative traces of neurons expressing RFP-S3A or RFP (control) in isolated or non-isolated rats. Scale bars 25 pA, 20 ms. Social isolation decreased the AMPA/NMDA ratio in neurons expressing RFP (isolated, n = 16; non-isolated, n = 23). The reduced AMPA/NMDA ratio was prevented in neurons expressing RFP-S3A (isolated, n = 15; non-isolated, n = 20). *P < 0.05 (ANOVA, *F*_(3,70)_ = 2.877). Error bars represent SEM. (**b**) Representative traces of neurons expressing HSV-S3A-IRES-Venus or HSV-IRES-Venus (control) from isolated or non-isolated rats. Scale bars 25 pA, 20 ms. S3A was expressed after the isolation period. Social isolation decreased the AMPA/NMDA ratio in neurons expressing HSV-IRES-Venus (isolated, n = 7; non-isolated, n = 11). The reduced AMPA/NMDA ratio was rescued in the neurons expressing HSV-S3A-IRES-Venus (isolated, n = 5; non-isolated, n = 6). *P < 0.05 (ANOVA, *F*_(3,25)_ = 4.550). Error bars represent SEM.

We recently reported that neonatal isolation-induced inactivation of ADF/cofilin disrupts the experience-driven synaptic delivery of the AMPA receptor in the juvenile mPFC^5^. Therefore, we examined here the effect of neonatal isolation-induced inactivation of ADF/cofilin on synaptic functions in the developing barrel cortex. We introduced expression vectors for GFP-actin and either RFP-ADF/cofilin Ser3A or RFP alone into the barrel cortex by *in utero* electroporation and subsequently exposed pups to the isolation procedure from P7-P11. We then prepared acute brain slices at P14 and recorded the synaptic transmission of pyramidal synapses at layer 4 to layer 2/3 of the barrel cortex. We measured the ratio of AMPA receptor-mediated transmission to N-methyl-D-aspartate (NMDA) receptor-mediated transmission (A/N ratio). We found that the A/N ratio of isolated rats expressing RFP-ADF/cofilin Ser3A was significantly greater than that of isolated rats expressing RFP alone, but comparable to that of non-isolated controls. This observation indicates that the isolation-induced disruption of experience-driven synaptic delivery of the AMPA receptor is mediated by ADF/cofilin inactivation (Fig. 3a).

Next, we asked if activation of ADF/cofilin after the isolation procedure leads to a recovery of synaptic delivery of the AMPA receptor. For that, we exposed pups to the isolation procedure from P7-P11, and then injected Herpes simplex virus expressing Venus-IRES-ADF/cofilin Ser3A or Venus-IRES alone into layer 2/3 of the barrel cortex at P13. Acute brain slices were prepared at P14 and synaptic transmission of pyramidal synapses at layer 4 to layer 2/3 of the barrel cortex was recorded. We found that the A/N ratio of isolated rats expressing Venus-IRES-ADF/cofilin Ser3A was significantly greater than that of isolated rats expressing Venus-IRES alone, but comparable to that of non-isolated controls (Fig. 3b). These results indicate that the neonatal isolation stress-induced inactivation of signalling pathways required for AMPA receptor trafficking can be recovered by the reactivation of ADF/cofilin.

### Social-isolation-induced increase in the stable actin fraction suppresses the experience-driven synaptic delivery of AMPA receptors in the developing barrel cortex

**Figure 4:**
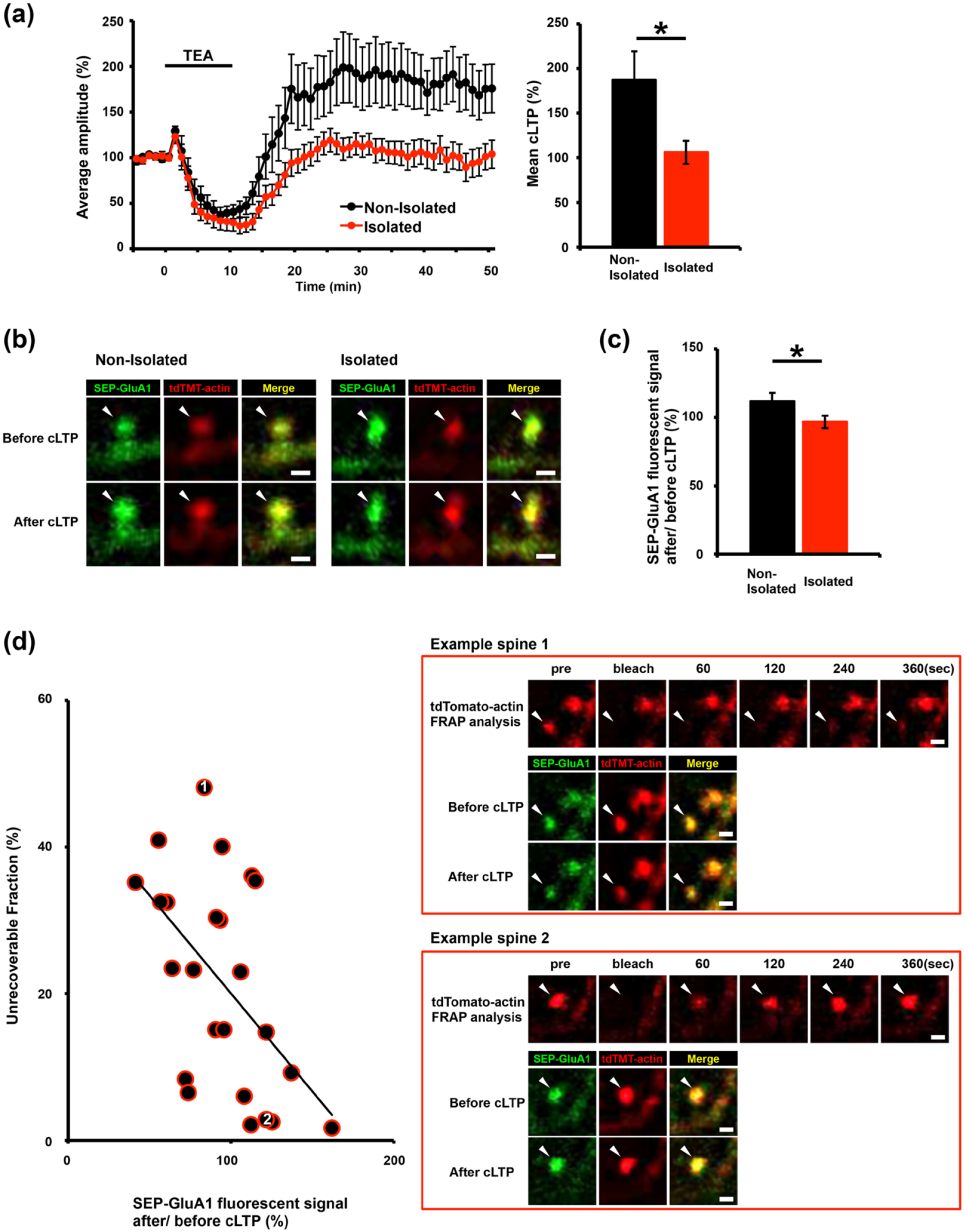
Disrupted GluA1 trafficking to the surface of spines in socially isolated rats is correlated with the increase in the stable actin fraction. (**a**) LTP was successfully induced in the barrel cortex of non-isolated rats (n = 13) by 10 min TEA treatment (black line). In contrast, chemical LTP (cLTP) induction was prevented in the barrel cortex of isolated rats (n = 10). (**b**) Representative images showing SEP-GluA1 and tdTomato-actin fluorescence on spines before and after TEA treatment in the barrel cortex of isolated or non-isolated rats. (**c**) Quantification of the changes in the SEP-GluA1 spine/dendrite ratio after TEA treatment. The integrated intensity of SEP-GluA1 fluorescent signal after/before cLTP on the spines was increased in non-isolated controls (n = 80) but not in isolated rats (n = 70). (**d**) Negative correlation between the surface expression of SEP-GluA1 at spines and the unrecoverable actin fraction at spines in the barrel cortex slices obtained from isolated rats. Two representative spine images: one with a high unrecoverable actin fraction and no cLTP-induced increase in the surface expression of SEP-GluA1 (Example spine 1) and a second with a low unrecoverable actin fraction and a cLTP-induced increase in the spine surface expression of SEP-GluA1 (Example spine 2). The scatter plot shows the changes in SEP-GluA1 expression (presented as a spine/dendrite ratio) after/before TEA treatment versus the fraction of unrecoverable tdTomato-actin at each of the individual spines. The fraction of unrecoverable actin was negatively correlated with the enhancement in surface GluA1 expression after/before cLTP in the barrel cortex slices obtained from isolated rats (*r* = 0.536, P < 0.05, n = 24). Representative images shown in the figures were Gaussian- and unsharp mask-filtered. Arrowheads indicate dendritic spines. Scale bar represents 1 µm. *P < 0.05 (Unpaired Student’s t-test). Error bars represent SEM.

We recently reported that the increased stable actin suppresses AMPA receptor trafficking at the mPFC synapses of isolated rats^5^. To investigate the relationship between the increased stable actin fraction and the suppression of AMPA receptor trafficking in the developing barrel cortex of isolated rats, we co-introduced expression vectors for super-ecliptic pHluorin (SEP) fused to the N-terminus of GluA1 and tdTomato-tagged actin into the rat barrel cortex by *in utero* electroporation. We then isolated rat pups as described above, and prepared acute brain slices at P14. The expression of SEP-GluA1 is selectively detected at the cell surface due to the strong fluorescence of SEP at or above pH 7 and the decreased fluorescence of SEP-GluA1 when localized to intracellular acidic secretory compartments. In the brain slices, we performed FRAP analysis to observe the tdTomato-actin dynamics at individual spines at layer 2/3 of the barrel cortex. We then induced chemical LTP by briefly exposing the slices to the potassium channel blocker tetraethylammonium (TEA). This method successfully induced LTP at of layers 4-2/3 synapses in the barrel cortical slices obtained from non-isolated rats but not from isolated rats (Fig. 4a). Consistent with this finding, chemically-induced LTP increased the surface expression of GluA1 in the spines in non-isolated rats but not in isolated rats (Fig. 4b,c). Interestingly, we found a negative correlation between the amount of stable actin and the LTP-induced increase in surface GluA1 at individual spines of the isolated rats (Fig. 4d). These findings suggest that the isolation-induced increase in the stable actin fraction suppresses experience-driven synaptic delivery of the AMPA receptor.

## Discussion

We previously reported that neonatal social isolation disrupts experience-driven synaptic AMPA receptor delivery to synapses in the developing rat barrel cortex, resulting in impairment of somatosensory perception^4, 6^. Further, we recently reported that neonatal social isolation inactivated the ADF/cofilin and led to the increased stable actin fractions at dendritic spines in the juvenile mPFC^5^. Isolation-induced increase of stable actin fractions prevented the experience-driven synaptic delivery of AMPA receptors and resulted in increase of aggression, one of antisocial behaviour^5^. Recent studies suggest that aggressive behaviour related with hypoactivation of the PFC is thought to be associated with the dysregulation of somatosensory system^36^. Also, it is reported that the development of mPFC is affected by sensory stimuli early in life^30^. Thus, the integrative neuronal network between mPFC and somatosensory cortex might be required to establish social behaviours. Here, we found that the same molecular events occur at spines in the developing barrel cortex of animals with neonatal social isolation. The wide effects of neonatal isolation among multiple cortical areas might be important to understand the molecular mechanism of antisocial behaviour.

Actin turnover is required for the trafficking of AMPA receptors. Latrunculin A, an agent that inhibits actin polymerization by sequestering actin monomers, attenuates the chemical LTP-induced increase in AMPA receptor surface expression at spines^26^. Furthermore, jasplakinolide, a natural cyclic peptide that binds actin filaments to promote actin polymerization and inhibit depolymerization, thereby stabilizing actin filaments, also inhibits AMPA receptor trafficking to the plasma membrane induced by chemical LTP^26^. Phalloidin, which stabilizes actin filaments, attenuates the LTP magnitude^37^. Together with our current results and previous findings^5^, these data suggest that the increased fraction of isolation-induced stable actin detected in isolated animals may be the primary mechanism underlying the isolation-induced blockade of synaptic AMPA receptor delivery.

We recently found that neonatal social isolation leads to the activation of LIM kinase and inactivation of ADF/cofilin in the juvenile mPFC. Here, we observed the same signalling events in the developing barrel cortex of rats exposed to neonatal isolation. Thus, neonatal isolation could lead to widespread molecular alterations of common nature throughout the neocortex. Furthermore, we found that Rac1 activity was increased in the developing barrel cortex of isolated rats, which could result in the activation of Rac1, leading to the elevation of LIM kinase activity^35^. ADF/cofilin is inactivated by LIM Kinase mediated phosphorylation, while dephosphorylation of ADF/cofilin is regulated by chronophin phosphatase, slingshot phosphatase^38^, and protein phosphatase 1, 2A and 2C^39^. Slingshot and protein phosphatase 1 also have important roles in synaptic AMPA receptor trafficking by controlling actin cytoskeleton^40, 41^. Therefore, neonatal social isolation have potential to affect various ADF/cofilin regulatory pathways in the spine, resulting in the disruption of synaptic AMPA receptor delivery.

Recently, the effect of chronic stress on glutamate receptors has been reported in the various brain regions^42^. Early life stress decreased expression of hippocampal AMPA receptor subunits, GluA1 and GluA2, and NMDA receptor subunits, GluN1^43^. Chronic stress in adult mice also decreased the total and surface protein levels of NMDA receptor subunits as well as AMPA receptors in the PFC^44^. In the basolateral amygdala, chronic stress increased GluA1 phosphorylation at Ser 845 and induced calcium-permeable AMPA receptors^45^. The protein levels of GluN1 subunit of the NMDA receptor was also upregulated in the amygdala of rats exposed to the chronic stress^46^. In the nucleus accumbens (NAc) of adult mice, chronic social stress was reported to increase the protein level of GluA1^47^ and reduce Rac1 expression via epigenetic regulation^48^. Thus, chronic stress exhibited various effects on AMPA receptors and NMDA receptors in multiple brain regions. According to previous reports and our data^5^, chronic stress could lead to neocortical and limbic dysfunction through multiple Rac1-ADF/cofilin mediated mechanisms, leading to disruption of synaptic AMPA receptor delivery. The imbalance of the activity among the interconnected brain regions might cause various symptoms of human psychiatric disorders.

## Methods

### Ethics Statement

All experiments were conducted according to the Guide for the Care and Use of Laboratory Animals (Japan Neuroscience Society) and the Guide for the Yokohama City University. All animal experiments were approved by the Animal Care and Use Committee of Yokohama City University (authorization number: F-A-14-028). All surgical procedures were performed under anesthesia, and every effort was made to minimize suffering.

### Animals and neonatal social isolation

Sprague-Dawley (SD) rats (Charles River Laboratories), and multiple colonies that contained five males and five females each were used. Rats were housed in plastic ekon cages and maintained on a 14 h light/10 h dark cycle (full light at 0500 and full darkness at 1900). The temperature and humidity were held constant at 22 °C ± 1 °C and 55% ± 5%, respectively. Food and water were provided ad libitum. Procedures were performed in strict compliance with the animal use and care guidelines of Yokohama City University.

During the neonatal social isolation procedure, three male pups were isolated from their mother and siblings for six hours per day from 10:00 to 16:00 between postnatal day 7 (P7) and P11. During the isolation period, each male pup was kept alone in a smaller cage placed over a heating pad at 35 °C and moved into an adjacent room.

### Constructs

GFP-actin and tdTomato-actin were PCR amplified and subcloned into the pCAGGS-EX and pEF-BOS vectors, respectively. pCALNL-GluA1 and pCAG-ERT2CreERT were gifts from R. Malinow (University of California at San Diego). The Cofilin Ser3A cDNA-mRFP construct was a gift from J. Zheng (Emory University School of Medicine). The Cofilin Ser3A cDNA-mRFP was PCR amplified and subcloned into the pEF-BOS and HSV-IRES-Venus vectors.

### ***In Utero*** Electroporation

Layer 2/3 progenitor cells were transfected by *in utero* electroporation. Embryonic day 17 (E17) pregnant SD rats were anesthetized with an isoflurane-oxygen mixture. Approximately 0.5 ml of DNA solution containing fast green was pressure injected through a pulled-glass capillary tube by mouth into the left lateral ventricle of each embryo. The head of each embryo was placed between tweezer electrodes with the anode contacting the left hemisphere. Electroporation was achieved with five square pulses (duration = 50 ms, frequency = 5 Hz, voltage = 80 V; BEX Co.).

### ***In vivo*** infection of barrel cortex neurons

Rats were deeply anesthetized with an isoflurane-oxygen mixture. The skin covering the skull was cut and gently pushed to the side. The anterior fontanel was identified and a region 2-mm posterior, 4.5-mm lateral was gently pierced with a dental drill. The recombinant Herpes virus was pressure-injected through a pulled-glass capillary (Narishige) into the barrel cortex. After injection, the skin was repositioned and its integrity was maintained with cyanoacrylate glue. Rats were kept on a heating pad during the procedures, and returned to their home cage after regaining movement.

### Analysis of FRAP data

Images were obtained using a two-photon laser-scanning microscope (FV-1000MPE; Olympus) with a water immersion objective (25 × 1.05 NA; Olympus). FRAP analysis was performed using a macro function of the stimulus setting menu in the Fluoview software to control sequential image acquisition and emission of a photobleaching laser pulse to the region of interest (ROI). A single dendritic spine of a layer 2/3 neuron in the rat barrel cortex was set as the ROI and two pre-bleaching images acquired. The spine fluorescence was then photobleached with a two-photon laser at 910 nm. The recovery of fluorescence was traced for an additional 6 min by acquiring images at 20-s intervals. Minimum laser power was used to prevent photobleaching during the pre- and post-bleaching stages. Background fluorescence was subtracted from the fluorescence of the target spine. The intensity of bleached spines was normalized to the baseline fluorescence and normalized to neighbouring non-photobleached spines at each time point. The GFP-actin signals were fitted to a single exponential curve using the following equation (Igor Pro, Wavemetrics):

$$F={F}_{t=\infty }+A\,exp(-\frac{t}{\tau })$$

where *F*_*t*=*∞*_ represents the unrecoverable fluorescence, considered to be a fixed population of fluorescent protein, and *τ* is the time constant for recovery.

### Western blotting

Barrel cortex samples were rapidly dissected and stored at −80 °C. Synaptoneurosome fractions were prepared as previously described^4^. Frozen samples were homogenized in ice-cold homogenization buffer (10 mM Hepes, 1.0 mM EDTA, 2.0 mM EGTA, 0.5 mM DTT, 0.1 mM PMSF, 10 mg/L leupeptin, and 100 nM microcystin). Tissue was homogenized in a glass/glass tissue homogenizer, and homogenates were passed through two 100-µm-pore nylon mesh filters, and then through a 5-µm-pore filter. Filtered homogenates were centrifuged at 3600 × g for 10 min at 4 °C. The resulting pellets were re-suspended in 100 µL of boiling homogenization buffer with 1% SDS, followed by immunoblotting. The signal intensity of each band was measured by MultiGauge (Fujifilm). The net signal was calculated by subtracting the background signal obtained from the region adjacent to the band^49^.

### Rac activation assay

The activation of Rac1 was measured with the Rac1 activity assay kit (Millipore) according to the manufacturer’s instructions. Briefly, a fusion protein consisting of the p21-binding domain of Pak1, which specifically binds to the active form of Rac1 (Rac-GTP), and not to the inactive form of Rac1 (Rac-GDP), was used for precipitation of Rac-GTP. This was followed by western blotting with the Rac1 antibody.

### Antibodies

Antibodies to phospho-Cofilin (Abcam: ab12866), Cofilin (Abcam: ab42824), phospho-LIM kinase (Cell Signaling Technology: 3841), LIM kinase (Cell Signaling Technology: 3842), Rac1 (Millipore: 05-389), and GAPDH (Cell Signaling Technology: 2118) were used.

### Drug treatment

Systemically-administered drugs were injected subcutaneously. RU486 (40 µg/g of body weight; Sigma-Aldrich) was dissolved in DMSO and injected twice a day during isolation.

### Electrophysiology

Rats were anesthetized with an isoflurane-oxygen mixture and the brains were removed. The brains were quickly transferred into gassed (95% O2 and 5% CO2) ice-cold dissection buffer as described previously^4^. Coronal brain slices were cut (350 µm, Leica VT1000) in dissection buffer. Slices were then incubated in artificial cerebrospinal fluid (ACSF) as described previously^50^.

Patch recording pipettes (3–7 MΩ) were filled with intracellular solutions as described previously^4^. The recording chamber was perfused with ACSF containing 0.1 mM picrotoxin, and 4 µM 2-chloroadenosine at 22–25 °C. Whole-cell recordings were obtained from layer 2/3 pyramidal neurons of the rat barrel cortex with a Multiclamp 700B (Axon Instruments). Bipolar tungsten stimulating electrodes were placed in layer 4. The stimulus intensity was increased until a synaptic response with an amplitude >~10 pA was recorded. Synaptic AMPA receptor-mediated currents at −60 mV and +40 mV were averaged over 50 trials. The AMPA/NMDA ratio was calculated as the ratio of the peak current at −60 mV to the current at +40 mV 50 ms after stimulus onset.

### Cre Recombinase Activation by 4-hydroxytamoxifen

4-hydroxytamoxifen (4-OHT; Sigma-Aldrich) was dissolved in ethanol at 20 mg/mL and diluted with 9 volumes of sesame oil (Sigma-Aldrich). Rats received intraperitoneal injections of diluted 4-OHT (2 mg/mL) at P12 (100 µl per animal).

### Chemically induced LTP

For chemical stimulation, brain slices were incubated in ACSF at room temperature, followed by stimulation with 25 mM TEA (Sigma) in ACSF for 10 min before changing back to regular ACSF. EPSCs were recorded for 40 min after the replacement with regular ACSF. Throughout the recording, EPSC amplitude was always normalized to the average baseline amplitude before stimulation.

### Imaging

Images were captured before and 30 min after TEA cLTP induction using a two-photon laser-scanning microscope (FV-1000MPE; Olympus) with a water immersion objective (25 × 1.05 NA; Olympus). SEP and tdTomato were excited at 910 nm with a Ti:sapphire laser (Mai Tai DeepSee; Spectra-Physics). Green and red fluorescent signals were separated by a set of dichroic mirrors and filters (Olympus). The SEP and tdTomato fluorescence in spines and dendrites was measured as integrated green and red fluorescence, respectively, after background and leak subtraction. The ratio of the SEP fluorescence intensity of the spine head to the dendritic shaft was measured on manually selected spine head and dendritic shaft areas.

### Statistics

All data are expressed as the mean ± standard error of the mean (SEM). Experiments with two groups were statistically analysed using unpaired Student’s t-tests. Experiments with more than two groups were subjected to one-way ANOVA followed by Fisher’s protected least significant difference (PLSD) post-hoc test.

## Electronic supplementary material

Supplementary Figure 1
