# Supplementary material for: Social isolation suppresses actin dynamics and synaptic plasticity through ADF/cofilin inactivation in the developing rat barrel cortex
Source: Sci Rep. 2017 Aug 16;7:8471. doi: 10.1038/s41598-017-08849-3 (PMC5559554; doi:10.1038/s41598-017-08849-3)

Social isolation suppresses actin dynamics and synaptic plasticity through ADF/cofilin inactivation in the developing rat barrel cortex

Hirobumi Tada[1,2] Tomoyuki Miyazaki[1], Kiwamu Takemoto[1,3], Susumu Jitsuki[1], Waki Nakajima[1], Mayu Koide[1], Naoko Yamamoto[1], Akiko Taguchi[2], Honami Kawai[1], Kasane Komiya[1], Kumiko Suyama[1], Hiroki Abe[1], Akane Sano[1], and Takuya Takahashi[1*]

[1] Department of Physiology, Yokohama City University Graduate School of Medicine, Yokohama, 236-0004, Japan.

[2] Department of Integrative Aging Neuroscience, National Center for Geriatrics and Gerontology, Aichi, 474-8511, Japan

[3] JST, PRESTO, Saitama, 332-0012, Japan

Supplementary Figure 1

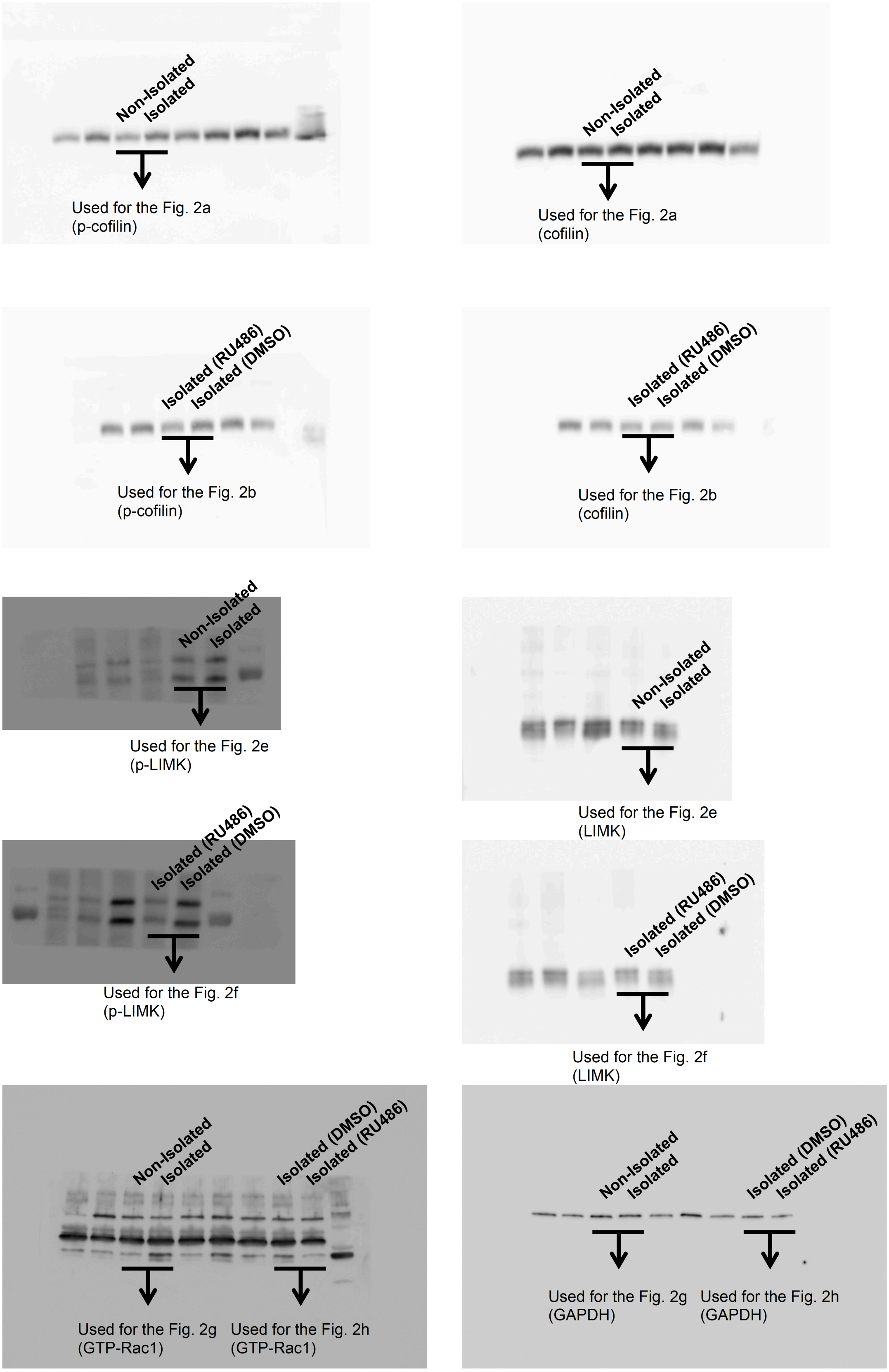

Supplement: Supplementary file 1 — Supplementary Figure 1 [file 41598_2017_8849_MOESM1_ESM.pdf]
